# Knowledge, perceptions, and management of cancer-related fatigue: the patients' perspective

**DOI:** 10.1007/s00520-020-05686-5

**Published:** 2020-08-29

**Authors:** Martina E. Schmidt, Susanne Bergbold, Silke Hermann, Karen Steindorf

**Affiliations:** 1 Division of Physical Activity, Prevention and Cancer, National Center for Tumor Diseases (NCT) and German Cancer Research Center (DKFZ), Im Neuenheimer Feld 581, 69120 Heidelberg, Germany; 2 Epidemiological Cancer Registry Baden-Württemberg, German Cancer Research Center (DKFZ), Heidelberg, Germany

**Keywords:** Fatigue management, Screening, Cancer survivorship, Exhaustion, Quality of life, Patient perspective

## Abstract

**Purpose:**

This study aimed to assess cancer patients’ knowledge and perceptions regarding fatigue and to provide up-to-date information on the current fatigue management from the patients’ perspective.

**Methods:**

The FiX study recruited 2508 cancer patients approximately 2 years after diagnosis via a cancer registry in Germany. Participants completed a questionnaire on their knowledge and perceptions of fatigue and the management received.

**Results:**

More than half of respondents (58%), especially among the elderly patients, did not feel well informed about fatigue. Overall, 41% reported having never been asked about being exhausted by their treating physician. Even fewer patients stated that general practitioners or nurses have asked if they felt exhausted. Only 13% of patients who had experienced severe fatigue had received a fatigue assessment by means of a rating scale or questionnaire—although this is recommended by existing guidelines for fatigue screening. Health care professionals seemed less likely to address fatigue with elderly as well as female patients. The most commonly reported measure against fatigue was exercise or regular physical activity (68%). However, this was mostly done on patients’ own initiative. Psychological support was rarely used (13%) and only in approximately half of the cases upon the advice of a physician. Yoga, another promising intervention against fatigue, was performed by only 9% of patients.

**Conclusions:**

Our study indicates deficits in terms of knowledge, education, screening, counseling, and treatment for fatigue and highlights starting points for targeted improvements in fatigue management based on patients’ needs.

**Electronic supplementary material:**

The online version of this article (10.1007/s00520-020-05686-5) contains supplementary material, which is available to authorized users.

## Introduction

Fatigue is one of the most common and burdensome symptoms experienced by cancer patients and may last for months and even years after end of cancer treatment [1]. Nevertheless, many cancer patients seem poorly educated about this problem. Despite being severely impaired, many patients do not even know that they are suffering from a syndrome that has a name and is potentially treatable. Lack of knowledge about the experienced symptoms can cause anxiety and unnecessary fear of disease progression. Thus, conveying the knowledge that these symptoms are common among cancer patients, often transient, potentially treatable, and are not an indicator for tumor progression may provide patients with a sense of control and is a prerequisite to seek for help. Information and education can also promote skills and motivation for behavior changes that may support coping with or alleviating fatigue [2, 3].

Likewise, providing information and education about cancer-related fatigue is recommended by the US National Comprehensive Cancer Network (NCCN) [4], the Canadian Association of Psychosocial Oncology (CAPO) [5], as well as by the European Society for Medical Oncology (ESMO) [6]. This should take place prior to cancer treatment, especially if fatigue is a known and frequent side effect of the administered therapy. Furthermore, education about fatigue should be provided repeatedly for all cancer patients irrespective of type of treatment, during as well as after therapy. A Cochrane review indicated that educational interventions for the management of cancer-related fatigue may have a moderate effect on reducing fatigue distress and may help reduce anxiety [3].

Although education about fatigue can be found in the Internet and numerous well-written leaflets exist, this information often does not reach the patients. On the part of the health care professionals (HCP), the minimum necessary step would be to ask each patient if he/she feels exhausted. Patients may not address fatigue symptoms by themselves. Some patients believe that exhaustion is an unavoidable consequence of cancer, some may fear that the optimal treatment dosage could be reduced if they report side effects, and others may just not get the chance to appropriately describe this subjective feeling of exhaustion due to lack of time or due to communication barriers.

While querying and explaining fatigue is a fundamental first step, further support needs to be provided by HCPs. The currently recommended fatigue management comprises (1) routine screening at the initial visit, at regular follow-up intervals, and whenever clinically indicated; (2) in case of a positive screening result: diagnostic assessment of the characteristics, impacts, and potentially treatable contributing factors or causes of fatigue; (3) counseling and support to take appropriate actions or therapies based on the focused evaluation; and (4) re-evaluation to check therapy success [1, 5–7]. Previous studies, however, indicated that the implementation of these guidelines into practice is a challenge and not yet sufficiently established [8, 9].

Thus, we aimed to provide up-to-date information on the patients’ experience with the current fatigue management in Germany with regard to their knowledge and perceptions about fatigue, what steps have been provided, and by whom and which actions were taken and to identify deficits or starting points for targeted improvements in fatigue management based on patients’ needs.

## Methods

### Study population

Between March 2018 and May 2019, the FiX study recruited patients approximately 2 years after a primary cancer diagnosis. Patients were randomly sampled from the Epidemiological Cancer Registry of Baden-Württemberg, Germany, stratified by entity, gender, and age group. The Trust Center of the Cancer Registry sent letters to a total of 11,113 patients asking for permission to transfer their contact data to the FiX study group. Of those, 1277 (11.5%) patients were not reached (i.e., due to invalid/unknown address) and 1415 (12.7%) were already deceased. Thus, 8421 patients were contacted, of which 2508 (29.8%) gave informed consent to participate, 2694 (32.0%) actively refused data transfer to the study center, and 243 (2.9%) who agreed to the transfer of contact data did not return the questionnaires, whereas of the remaining 2976 (35.3%) patients, no feedback was received.

### Assessments

After written informed consent, patients were asked to complete a short questionnaire online or on paper, including the following question: “During your cancer treatment, were you asked whether and to what extent you felt exhausted?”. Patients should tick whether they were asked about exhaustion/fatigue (1) not at all, (2) only briefly and generally, or (3) whether a more detailed assessment of fatigue was performed, e.g., using a 0–10 scale or a questionnaire. The question was separately asked with regard to the treating physician, nurses, general practitioners, psychosocial counseling services, and “other (please specify).” Further, along a checklist with potential actions, patients were asked: “Which of the offers or measures listed below have you used since cancer diagnosis? Please tick on whose initiative/advice you have taken this measure (action not taken; taken on own initiative; taken upon advise/mediation of physician, nurse, friend/relative, other).” Multiple answers were possible. To assess knowledge and perceptions, patients should state their agreement with seven statements on a 5-point Likert scale (1 = do not agree at all; 5 = fully agree).

Usual fatigue in the last 24 h and usual as well as maximal fatigue since cancer diagnosis (retrospectively) were assessed on a 0–10 scale. For stratified analysis, patients were categorized as having ever experienced severe (score ≥ 8), moderate (5–7), or only mild (0–4) fatigue based on the score of maximal fatigue experienced since diagnosis. Previous and current cancer treatment was recorded by the patients and supplemented by data from the cancer registry.

### Statistical methods

Responses are presented by descriptive statistics. In addition, we explored which factors determined that HCPs addressed fatigue using multiple logistic regression models. Following factors were a priori considered potential determinants and simultaneously included in the models: sex, age, BMI, chemo-, radio-, targeted, and endocrine therapy, surgery, time since diagnosis, and tumor entity. As the number of missing values in those covariates were low (< 2%), we used complete cases.

## Results

Characteristics of the included 2508 cancer survivors are presented in Table 1. The participants were included on average 2.0 ± 0.8 years after cancer diagnosis with a mean age of 66.0 ± 11.9 years and a mean body mass index (BMI) of 26.8 ± 5.5. On average, the maximal experienced fatigue since diagnosis was rated as 6.2 ± 3.0 on a 0–10 scale. The distribution of maximal fatigue by tumor entity is presented in supplemental figure S1.

**Table 1:** Characteristics of study population (*N* = 2508)

Characteristic		Mean	(SD)
Age		66.0	(11.9)
BMI (kg/m^2^)		26.8	(5.5)
Years since diagnosis		2.0	(0.8)
Usual fatigue before diagnosis (0–10 scale)		2.5	(2.1)
Usual fatigue in last 24 h (0–10 scale)		3.4	(2.3)
Maximal fatigue since diagnosis (0–10 scale)		6.2	(3.0)
		*N*	**%**
Primary tumor	Breast	232	9.3
	Prostate	223	8.9
	Multiple primaries	212	8.5
	Non-Hodgkin lymphoma	210	8.4
	Kidney	207	8.3
	Rectum	195	7.8
	Colon	189	7.5
	Endometrium	176	7.0
	Malignant melanoma	173	6.9
	Leukemia	158	6.3
	Ovaries or cervix	150	6.0
	Bladder	140	5.6
	Stomach	125	5.0
	Lung	37	1.5
	Pancreas	33	1.3
	Liver	30	1.2
	Other	18	0.7
Sex	Male	1262	50.3
	Female	1225	48.8
	Missing	21	0.8
BMI	Underweight (< 18.5)	44	1.8
	Normal (18.5–< 25)	962	38.4
	Overweight (25–< 30)	892	35.6
	Obese (30–< 35)	374	14.9
	Severe obese (≥ 35)	161	6.4
	Missing	75	3.0
Chemotherapy^a^	Never	1439	57.4
	In the past	911	36.3
	Recent/current	132	5.3
	Missing	26	1.0
Radiotherapy^a^	Never	1779	70.9
	In the past	685	27.3
	Recent/current	24	1.0
	Missing	20	0.8
Targeted therapy^a^	Never	1999	79.7
	In the past	361	14.4
	Recent/current	125	5.0
	Missing	23	0.9
Endocrine therapy^a^	Never	2069	82.5
	In the past	262	10.4
	Recent/current	155	6.2
	Missing	22	0.9
Cancer surgery^a^	Never	410	16.3
	In the past	2019	80.5
	Recent/current	38	1.5
	Missing	41	1.6

^a^Recent/current, within the last 4 weeks; in the past, more than 4 weeks ago

Table 2 summarizes the responses to the question “During your cancer treatment, were you asked whether and to what extent you felt exhausted?”. Overall, 41.0% of patients did not recall to be ever approached by their treating physician with regard to exhaustion, whereas 47.9% had been asked about exhaustion, however, only with a brief question. Only for 7.1% of patients, fatigue was assessed by a treating physician in more detail, e.g., using a screening scale, questionnaire, or longer talk. Fatigue was addressed even less by general practitioners and scarcely by nurses. Psychosocial services—in absolute terms—did not contribute substantially to addressing fatigue. However, these responses need to be interpreted differently than those of the first three health professions, because only a minority of patients might have had contact to psychosocial services. Only 173 patients reported having been asked about exhaustion by other persons, reporting as most common answer (*n* = 64) therapists/personnel in the stationary rehabilitation that is offered in Germany for cancer patients, followed by relatives or friends (*n* = 52). In the bottom rows of Table 2, the responses for the subgroup of patients who had experienced severe fatigue since diagnosis (score ≥ 8 on a 0–10 scale) are presented. Among these patients, fatigue had been addressed slightly more often than among patients who had experienced only moderate or little/no fatigue (Chi^2^ *p* < 0.001 for each health care profession). Still, more than a quarter of these patients with severe fatigue were not approached by any HCP regarding this burdensome symptom, and only about 13% of these patients had received an in-depth assessment of their prevailing problem.

**Table 2:** Proportions of patients reporting that health care professionals asked them about fatigue

Exhaustion/fatigue addressed^a^	Not at all		Short question		More detailed, e.g., using a rating scale		Don’t know	
	*N*	%	*N*	%	*N*	%	*N*	%
All patients								
Treating physician	984	41.0	1150	47.9	171	7.1	98	4.1
General practitioner	1235	54.3	885	38.9	72	3.2	83	3.7
Nurse	1553	73.1	388	18.3	54	2.5	130	6.1
Psychosocial service	1741	84.1	174	8.4	52	2.5	103	5.0
By any of the above listed HCPs	811	33.2	1301	53.3	235	9.6	95	3.9
Patients who experienced severe fatigue since diagnosis								
Treating physician	370	35.6	542	52.2	93	9.0	34	3.3
General practitioner	473	47.7	446	45.0	40	4.0	32	3.2
Nurse	646	69.6	200	21.6	33	3.6	49	5.3
Psychosocial service	732	80.7	96	10.6	37	4.1	42	4.6
By any of the above listed HCPs	283	26.7	615	58.0	134	12.6	29	2.7

^a^Numbers and percentages based on non-missing answers

Regression analyses showed that treating physicians, general practitioners, and nurses addressed fatigue less frequent among elderly patients compared with patients below 70 years of age (Table 3). Fatigue seemed to be addressed significantly more often by treating physicians as well as nurses if patients received or were currently receiving chemotherapy (ORs between 1.8 and 4.9). Patients with liver cancer (OR 6.4) and leukemia (OR 2.7) showed significantly higher odds than breast cancer patients for having been asked about exhaustion/fatigue by their treating physician. Also liver (OR 3.2), pancreas (OR 2.9), and colon (OR 2.0) cancer patients were more frequently asked about exhaustion by their general practitioner than breast cancer patients. Even adjusted for cancer type and treatment, physicians and nurses seemed somewhat less likely to address fatigue with female patients compared with male (OR 1.3 and 1.4, respectively).

**Table 3:** Logistic regression results on factors associated with addressing fatigue by health professionals

		Exhaustion/fatigue was at least briefly addressed by a:		
		Treating physician	General practitioner	Nurse
		OR (95% CI)	OR (95% CI)	OR (95% CI)
Sex	Female	1.0 (Ref.)	1.0 (Ref.)	1.0 (Ref.)
	Male	*1.3 (1.0, 1.7)*	1.1 (0.8, 1.3)	*1.4 (1.0, 1.8)*
Age	< 60 years	1.0 (Ref.)	1.0 (Ref.)	1.0 (Ref.)
	60–70 years	*0.8 (0.6, 1.0)*	0.9 (0.7, 1.1)	0.9 (0.7, 1.2)
	≥ 70 years	*0.5 (0.4, 0.6)*	*0.7 (0.6, 0.9)*	*0.5 (0.4, 0.7)*
BMI	Obese (≥ 30)	1.0 (Ref.)	1.0 (Ref.)	1.0 (Ref.)
	Overweight (25–< 30)	1.0 (0.8, 1.3)	0.9 (0.7, 1.2)	0.9 (0.7, 1.3)
	Normal (18.5–< 25)	1.1 (0.8, 1.4)	0.8 (0.7, 1.1)	1.0 (0.7, 1.3)
	Underweight (< 18.5)	1.7 (0.8, 3.7)	0.9 (0.4, 1.9)	2.0 (0.8, 5.0)
Chemotherapy^a^	Never	1.0 (Ref.)	1.0 (Ref.)	1.0 (Ref.)
	In the past	*1.8 (1.4, 2.2)*	1.1 (0.9, 1.5)	*2.8 (2.1, 3.8)*
	Recently/currently	*2.4 (1.5, 3.9)*	1.1 (0.7, 1.7)	*4.9 (2.9, 8.1)*
Radiotherapy^a^	Never	1.0 (Ref.)	1.0 (Ref.)	1.0 (Ref.)
	In the past	*1.5 (1.1, 1.9)*	1.1 (0.8, 1.4)	1.0 (0.7, 1.4)
	Recently/currently	1.1 (0.4, 2.9)	2.0 (0.8, 5.1)	1.7 (0.5, 5.3)
Targeted therapy^a^	Never	1.0 (Ref.)	1.0 (Ref.)	1.0 (Ref.)
	In the past	*1.5 (1.1, 2.0)*	1.0 (0.7, 1.4)	1.1 (0.8, 1.6)
	Recently/currently	1.5 (0.9, 2.3)	1.0 (0.6, 1.5)	1.2 (0.7, 1.9)
Endocrine therapy^a^	Never	1.0 (Ref.)	1.0 (Ref.)	1.0 (Ref.)
	In the past	1.0 (0.7, 1.5)	1.2 (0.8, 1.7)	0.9 (0.6, 1.5)
	Recently/currently	1.5 (0.9, 2.6)	0.8 (0.5, 1.3)	0.7 (0.4, 1.4)
Surgery^a^	Never	1.0 (Ref.)	1.0 (Ref.)	1.0 (Ref.)
	In the past	1.0 (0.7, 1.4)	1.2 (0.9, 1.7)	1.2 (0.8, 1.8)
	Recently	1.7 (0.7, 3.8)	1.4 (0.7, 3.1)	*3.8 (1.6, 9.2)*
Time since diagnosis	Per 1 year unit	0.9 (0.8, 1.1)	0.9 (0.8, 1.1)	0.9 (0.7, 1.1)
Entity	Breast	1.0 (Ref.)	1.0 (Ref.)	1.0 (Ref.)
	Bladder	0.7 (0.4, 1.2)	0.7 (0.4, 1.3)	*0.4 (0.2, 0.9)*
	Cervix	0.5 (0.2, 1.4)	1.4 (0.5, 4.0)	0.7 (0.2, 2.5)
	Colon	1.2 (0.7, 2.1)	*2.0 (1.1, 3.5)*	1.0 (0.5, 2.0)
	Endometrium	0.9 (0.5, 1.5)	0.8 (0.5, 1.3)	0.8 (0.4, 1.6)
	Kidney	0.7 (0.4, 1.2)	0.9 (0.5, 1.5)	*0.4 (0.2, 0.9)*
	Leukemia	*2.7 (1.4, 5.2)*	1.4 (0.7, 2.7)	1.1 (0.5, 2.4)
	Liver	*6.4 (2.0, 21)*	*3.2 (1.3, 8.2)*	2.0 (0.7, 5.9)
	Lung	1.2 (0.5, 2.8)	1.8 (0.8, 4.1)	0.9 (0.3, 2.4)
	Malignant melanoma	*0.6 (0.3, 1.0)*	*0.5 (0.3, 1.0)*	0.5 (0.2, 1.1)
	Non-Hodgkin lymphoma	1.7 (0.9, 3.0)	1.4 (0.8, 2.4)	1.1 (0.6, 2.2)
	Other or multiple entities	1.0 (0.3, 3.7)	0.6 (0.2, 2.5)	1.8 (0.4, 7.7)
	Ovaries	1.4 (0.8, 2.6)	1.5 (0.8, 2.7)	0.6 (0.3, 1.3)
	Pancreas	1.8 (0.7, 4.6)	*2.9 (1.2, 7.2)*	0.8 (0.3, 2.3)
	Prostate	1.1 (0.7, 1.9)	0.9 (0.5, 1.5)	*0.5 (0.2, 1.0)*
	Rectum	0.7 (0.4, 1.2)	1.0 (0.6, 1.8)	0.7 (0.3, 1.3)
	Stomach	1.1 (0.6, 2.1)	1.6 (0.8, 2.9)	0.5 (0.2, 1.1)

The logistic models simultaneously include all listed factors

^a^Recently/currently, within the last 4 weeks; in the past, more than 4 weeks ago

Significant results (*p* < 0.05) are written in italics

**Fig. 1:**
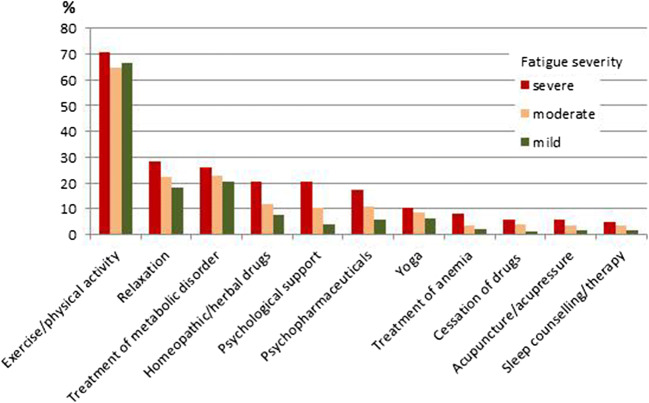
Offers or measures applied by patients, stratified according to fatigue severity

Table 4 presents the results regarding which actions were taken and by whom they were advised or initiated. Among the listed actions, exercise/physical activity was by far the most frequently reported action (67.7%). This action was mainly self-initiated. Only 13% of patients sought psychological support. Of these, 50% did this on advice or initiation by a physician. In Fig. 1, the actions taken are presented graphically stratified by level of maximally experienced fatigue.

**Table 4:** Which actions were taken and by whom were they advised or initiated

	Ever applied since diagnosis		If action was taken: By whom was it advised/initiated (multiple answers possible)^**a**^									
			Self-initiated		Advised or mediated by a physician		Advised or mediated by a nurse		Advised or mediated by friends/relatives		Advised or mediated by other persons	
	*N*	**%**^**c**^	*N*	**%**	*N*	**%**	*N*	**%**	*N*	**%**	*N*	**%**
Exercise/physical activity	1659	67.7	1484	89.6	408	24.6	34	2.1	138	8.3	84	5.1
Relaxation	585	23.9	392	72.5	160	29.6	15	2.8	34	6.3	50	9.2
Treatment of metabolic disorders	575	23.5	64	12.1	504	95.6	6	1.1	4	0.8	2	0.4
Homeopathic or herbal drugs	354	14.5	201	59.3	138	40.7	6	1.8	71	20.9	31	9.1
Psychological support	319	13.0	169	55.1	169	55.1	15	4.9	37	12.1	11	3.6
Psychopharmaceuticals	302	12.3	67	24.0	234	83.9	1	0.4	4	1.4	5	1.8
Yoga	211	8.6	184	87.2	29	13.7	2	1.0	16	7.6	13	6.2
Treatment of anemia	129	5.3	20	16.5	107	88.4	0	0.0	4	3.3	1	0.8
Cessation of drugs^b^	101	4.12	45	48.4	58	62.4	1	1.1	4	4.3	3	3.2
Acupuncture or acupressure	97	3.96	56	57.73	36	37.1	3	3.1	8	8.3	6	6.2
Sleep counseling or therapy	89	3.63	38	43.18	60	68.2	2	2.3	3	3.4	8	9.1

^**a**^Percentages based on the number of patients who responded on this question

^b^Cessation of drugs with tiredness as side effect

^**c**^Percentages based on the number of patients who provided any information on applied actions (*N* = 2446)

**Fig. 2:**
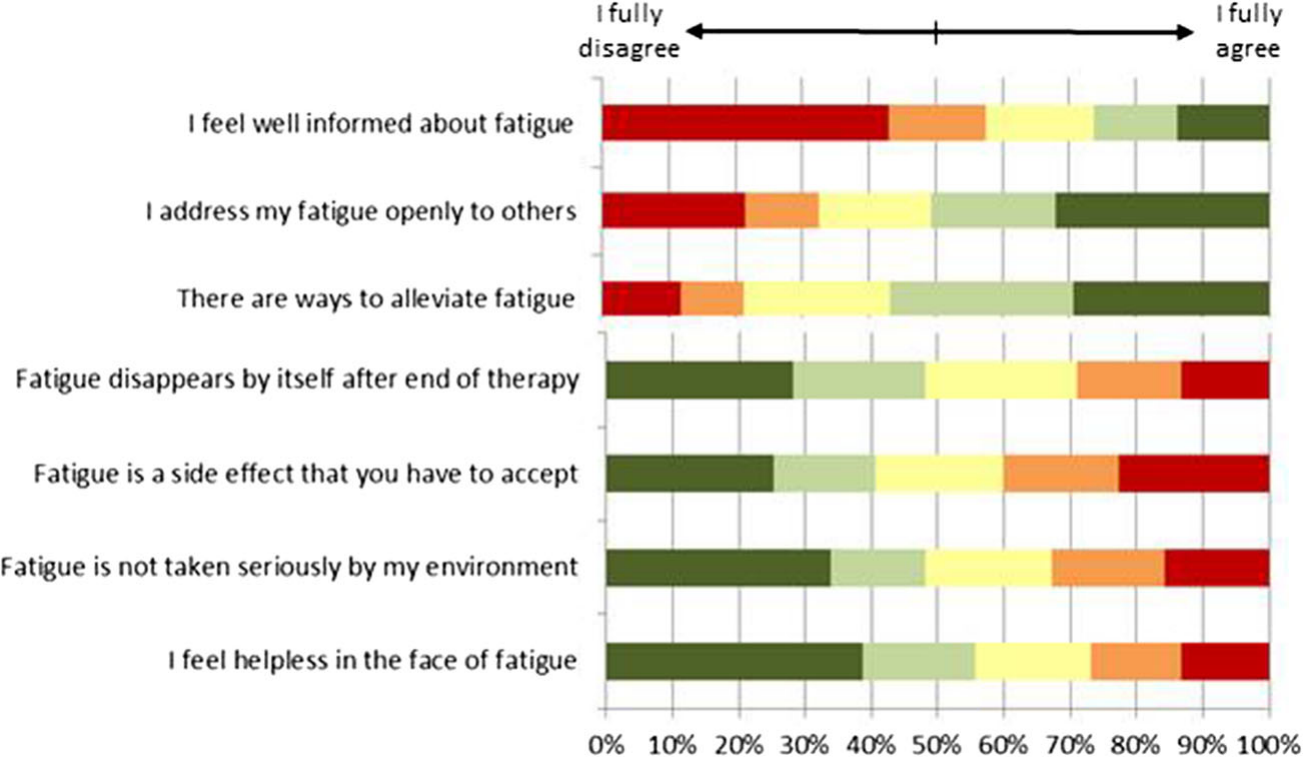
Patients’ knowledge and perceptions

Finally, Fig. 2 summarizes the responses on the questions regarding knowledge and perception of fatigue. Many patients skipped this last question on the FiX questionnaire or ticked the box “Don’t know”. Results in Fig. 2 are based on those who answered the questions (*n* = 1986). About 58% of responders fully or partly disagreed with the statement “I feel well informed about fatigue.” In addition, there are also beliefs and misconceptions about fatigue that could keep patients from seeking help, i.e., that there is no way to alleviate fatigue (21%); that fatigue is a side effect that has to be accepted (40%); or that fatigue disappears by itself after end of cancer treatment (29%); further barriers could be that 32% of responders do not address fatigue openly to others (friends, relatives, colleagues), 33% feel that fatigue is not taken seriously by their environment, and 27% feel helpless against fatigue.

Results differed significantly by age groups, with elderly patients more often feeling poorly informed (65% among age 70+ vs. 52% among age below 60 years) and believing that there is no way to alleviate fatigue (30% vs. 13%). Patients younger than 60 years more often stated that fatigue is not taken seriously by their environment (41%) compared with patients age 70+ (27%).

## Discussion

This survey among 2508 cancer survivors randomly sampled approximately 2 years after diagnosis indicates several deficits in the current fatigue management: (1) a lack of knowledge and education about fatigue, (2) a lack in systematic screening and diagnostic assessments of fatigue, and (3) insufficient counseling and referral to existing therapeutic offers.

### Knowledge and education

More than half of respondents, especially among the elderly patients, stated that they do not feel well informed about fatigue. Likewise, over a third of respondents have beliefs or attitudes about fatigue that could be barriers to address their symptoms towards HCPs and seek for help. Hence, health providers should address the topic and provide information pro-actively. Yet, our results show that patients often are not appropriately asked about fatigue symptoms. These results may be biased by poor recall. This is supported by the data showing that patients with current/recent cancer therapy are more likely to report being approached for exhaustion by a physician than patients who already completed cancer therapy more than 4 weeks ago. Nevertheless, even among patients who had experienced severe fatigue more than a third did not recall that this burdensome symptom was addressed by a physician, suggesting that the fatigue education and/or management—if any—was in many cases insufficient or too low level to be remembered. However, educating patients that physical, emotional, and cognitive exhaustions are common symptoms during and after cancer treatment, that these symptoms might indicate a syndrome that is known as “fatigue,” and asking them whether they have such symptoms is fundamental for patients to seek and/or receive appropriate prevention or treatment for fatigue.

Reasons for this shortcoming might be that treating physicians, general practitioners, and nurses themselves may not be well informed about fatigue and may feel helpless, in particular with regard to assistance and advice of how to ameliorate this symptom. There is no quick solution to this problem, for example, by prescribing medication. Moreover, many physicians and oncologists mainly focus on fighting the tumor and improving survival, considering fatigue as secondary since it is neither life-threatening nor requires immediate treatment. This is supported by a survey in 550 cancer patients, 400 oncologists, and 400 oncology nurses that showed that the oncologists and nurses substantially underestimated the prevalence and importance of fatigue [10]. Physicians and nurses are often already overloaded by clinical routine work and have too little time to provide additional information. Shortage of nurse human resources was identified as one important barrier to implement fatigue management in hospitals [11]. And quite essential, time spent for educating patients about fatigue is not adequately covered or refunded by the health insurance system. Moreover, even if fatigue has been explained as potential side effect, this information might not have reached the patient for various reasons: for example, the unknown technical term “fatigue” has been used, the patient was in an emotionally stressful situation, the patient was already overloaded with other information about cancer treatment, the patient was not yet affected by fatigue (e.g., pre-treatment), and the explanation was not adapted to the cognitive abilities or to language barriers.

### Screening and evaluation

Our results indicate that screening for fatigue, e.g., with a visual analogue scale, or further diagnostic assessments of fatigue symptoms have been scarcely performed. Even among patients who had experienced severe fatigue, less than 13% received such a more detailed assessment of their symptoms. Yet, these assessments are important as fatigue is a non-specific symptom, and potentially contributing factors such as anemia, pain, decreased functional status, comorbidities, or emotional distress need first to be clarified and symptomatically treated if possible. The results suggest that the current fatigue management is far from the actual guidelines of the NCCN, ESMO, or CAPO, recommending that each cancer patient should receive systematic and repeated screening for fatigue and—in case of indications for fatigue symptoms—comprehensive evaluations and diagnostic assessments.

The reason for this shortcoming probably lies at the institutional level. Systematic screening and evaluation of fatigue, as recommended by guidelines, need to be compulsory and integrated in clinical routine with clearly distributed responsibilities. This, so far, has been scarcely established.

### Counseling and therapy

Many randomized controlled trials have investigated interventions for fatigue. The current evidence is summarized in recent Cochrane reviews and other meta-analyses [1, 12–19]. Generally, non-pharmacological interventions have shown larger effects than pharmacological interventions [1, 13]. There is strong evidence that physical exercise, mind-body exercise (such as yoga, Tai-Chi, or Qigong), and specific psychosocial interventions have beneficial effects on cancer-related fatigue [3, 12, 15, 16, 18–23]. For other treatment approaches, e.g., bright white light therapy, acupuncture, or relaxation interventions, the evidence is still weak or inconclusive [6, 21, 24]. So far, it is unclear, which intervention may be most effective, but it is assumed that optimal treatment needs to be individually tailored based on an in-depth evaluation of the patient’s characteristics and fatigue pattern.

Our results show that a majority of patients reported having been physically active. This is generally positive, suggesting that many patients are at least aware that physical activity is beneficial. However, the self-reported activity may often be of low intensity or frequency, i.e., not reaching the level of supervised structured aerobic and/or resistance training interventions that have shown significant effects on fatigue in the randomized trials [25].

Patients who stated to have had experienced severe fatigue also had taken more measures than those with less or no fatigue. However, only a low proportion of affected patients have used approaches with good evidence for potential benefits, i.e., yoga by 10% and psychological support by 20%. These approaches seemed to be rarely recommended by HCP, possibly because they do not know about them or believe that many patients would not participate in Yoga or psychosocial interventions anyway. Hence, the potential of therapies has by long not been fully exploited.

### Perspectives for improvement of the fatigue management

In Germany, single clinics or cancer centers already work to achieve good fatigue management, yet, as our data show, it is still far from being implemented nationwide. These shortcomings in fatigue management do not exclusively exist in Germany. There are also reports from other countries suggesting that recommendations and guidelines for education, systematic screening, in-depth fatigue evaluation, and therapy of fatigue have not yet been implemented in practice [8, 9, 26, 27].

To overcome these problems, staff and financial resources may need to be allocated for fatigue education, and information about fatigue should become a systematic integrative part of clinical and supportive care. Hereby, information may need to be given repeatedly, at the beginning as well as during and after cancer therapy. It needs to be in plain language and adapted to the patients’ situation. Likewise, improving screening, evaluation, and therapy of fatigue may not be reached on an individual HCP level by advising the personnel to “make more effort in this regard.” Instead, a change of the health care system may be necessary, including compulsory guidelines as well as coverage by the health insurance system.

### Strengths and limitations

We cannot exclude a selection bias due to the low response rate. Yet, strong bias seems unlikely, because selection due to non-participation potentially was in two opposite directions: (1) patients with fatigue might have been too exhausted to participate, and (2) patients without fatigue might not have been interested to participate, because they were not affected by this problem. Further, data was self-reported, and recall bias cannot be excluded, likely resulting in an underestimation of the frequency with which HPCs have addressed fatigue problems. Nevertheless, even under a conservative assumption of about 20% false negative responses due to lack of recall, the results still indicate substantial shortcomings in knowledge, screening, and therapy. Fatigue might be more often addressed in the stationary rehabilitation, which is commonly after end of cancer therapy. This setting was however not in the focus of our survey. Strengths of the study include the large sample across a variety of common cancer entities, the systematic, representative sampling via a cancer registry, and the consideration of different aspects of fatigue management from the patients’ perspective.

## Conclusions

There is a clear lack of knowledge about cancer-related fatigue, and many patients feel poorly informed. It seems that many treating physicians do not address this problem enough or inadequately, lacking in-depth evaluations and diagnostic assessments of fatigue symptoms. Family doctors and nurses generally seem to consider fatigue symptoms even less. The potential of interventions with strong evidence for generally beneficial effects on fatigue has not yet been fully exploited. Systematic education, screening, diagnostic, counseling, and therapy for fatigue may need to be integrated as a structured and financially endowed component of routine care to ameliorate this frequent and burdensome symptom of cancer.

## Electronic supplementary material

ESM 1 (JPG 62 kb)
