# Supplementary figures and images for: Knowledge, perceptions, and management of cancer-related fatigue: the patients' perspective
Source: Support Care Cancer. 2020 Aug 29;29(4):2063–71. doi: 10.1007/s00520-020-05686-5 (PMC7892505; doi:10.1007/s00520-020-05686-5)

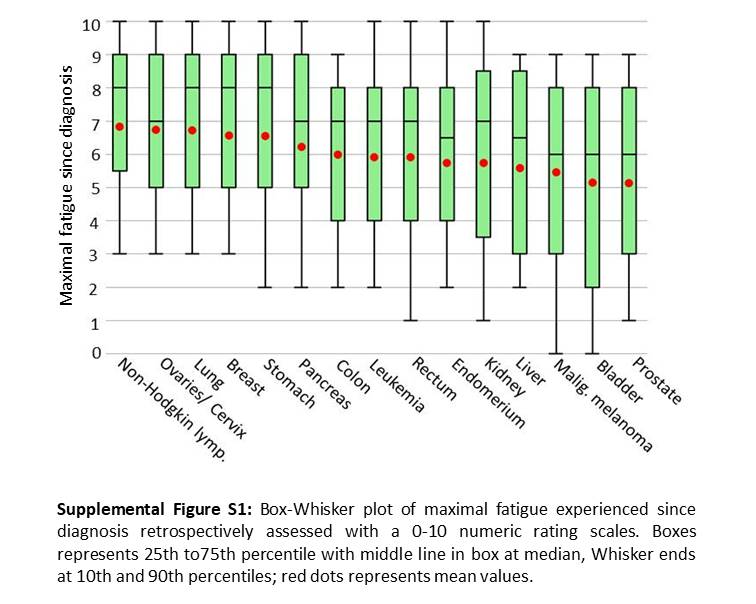

Supplement: Supplementary file 1 — (JPG 62 kb) [file 520_2020_5686_MOESM1_ESM.jpg]
